# Cancer Therapy-Associated Pulmonary Hypertension and Right Ventricular Dysfunction: Etiologies and Prognostic Implications

**DOI:** 10.31083/j.rcm2503087

**Published:** 2024-03-05

**Authors:** Orly Leiva, William Beaty, Steven Soo, Manyoo A. Agarwal, Eric H. Yang

**Affiliations:** ^1^Division of Cardiology, Department of Medicine, New York University Grossman School of Medicine, New York, NY 10016, USA; ^2^Department of Medicine, New York University Grossman School of Medicine, New York, NY 10016, USA; ^3^Department of Medicine, New York University Grossman Long Island School of Medicine, Mineola, NY 11501, USA; ^4^Cardio-Oncology Program, Heart, Vascular and Thoracic Institute, Cleveland Clinic Abu Dhabi, 00000 Abu Dhabi, United Arab Emirates; ^5^UCLA Cardio-Oncology Program, Division of Cardiology, Department of Medicine, University of California at Los Angeles, Los Angeles, CA 90095, USA

**Keywords:** cardio-oncology, cardiotoxicity, pulmonary hypertension, right ventricular dysfunction

## Abstract

Advances in cancer therapies have improved oncologic outcomes but can potentially expose patients to risk of cardiovascular toxicity. While left ventricular (LV) dysfunction is a well-known cardiotoxicity of cancer therapy. Pulmonary hypertension (PH) and right ventricular (RV) dysfunction are seen with several cancer therapies, including alkylating agents, tyrosine kinase inhibitors (TKIs), and immunotherapy, and are associated with significant morbidity and mortality. Awareness and recognition of cancer therapy-associated PH and RV dysfunction is critical to identify underlying etiologies and institute the appropriate therapy. However, gaps exist in the current literature on the epidemiology of PH and RV dysfunction in cancer, underlying pathophysiology and optimal management strategies.

## 1. Introduction 

Cancer is a leading cause of mortality worldwide but advances in cancer therapies have led to improved survival [1]. Cardiovascular toxicities associated with cancer therapies are increasingly recognized sources of morbidity and mortality among patients with cancer [2, 3, 4, 5]. Cardiomyopathy and myocardial injury are most associated with cancer therapies. However, pulmonary vascular disease, pulmonary hypertension and right ventricular (RV) dysfunction are a known but poorly understood form of cardiotoxicity stemming from certain cancer therapies [6]. While overall rare compared to other forms of known cardiotoxic manifestations of both historical and more modern cancer therapies, they can be associated with significant morbidity and mortality if not diagnosed; the spectrum of these findings are reviewed, along with imaging and treatment strategies [6, 7].

Pulmonary hypertension (PH) is hemodynamically defined by a mean pulmonary artery pressure (mPAP) of ≥20 mmHg at rest on right heart catheterization (RHC). Patients with PH may be categorized based on their hemodynamic characteristics as pre-capillary, isolated post-capillary (IpcPH), or combined pre- and post-capillary (CpcPH) [8]. Pre-capillary PH, which includes pulmonary arterial hypertension (PAH), is defined by a mPAP ≥20 mmHg, pulmonary capillary wedge pressure (PCWP) ≤15 mmHg and pulmonary vascular resistance (PVR) of >2 Wood units (WU). Post-capillary PH is defined as a mPAP ≥20 mmHg, PCWP >15 mmHg and PVR ≤2 and includes PH due to left-sided heart disease (including valvular pathology and heart failure). Combined pre- and post-capillary PH is defined as mPAP ≥20 mmHg, PCWP >15 mmHg and PVR >2 WU [8]. Post-capillary PH is primarily due to the transmission of increased left-sided pressures to the pulmonary vasculature and increasing pulmonary artery pressures a result. Pre-capillary PH is a pulmonary vasculopathy characterized by pathologic vasoconstriction, remodelling and fibrosis of the pulmonary arterioles leading to symptoms including dyspnea and eventually to RV failure [9].

Pulmonary hypertension is a clinically heterogenous disease characterized by increased pulmonary artery pressure. The clinical classification of PH focuses on the underlying cause of abnormal pulmonary artery pressure: Group 1 (PAH), Group 2 (left heart disease), Group 3 (due to lung disease and/or hypoxemia), Group 4 (chronic thromboembolic pulmonary hypertension), and Group 5 (unclear and/or multifactorial mechanisms including sickle cell disease and sarcoidosis) (Table 1) [10].

**Table 1.** **Clinical and hemodynamic classifications of pulmonary hypertension and implications in patients with cancer**.

WHO group	Mechanism	Hemodynamic classification	Sub classifications	Examples in cancer patients
1 – PAH	Vascular remodeling of pulmonary arterioles	Pre-Capillary	1.1 Idiopathic	Cancer therapy-induced PAH, PVOD
			1.2 Heritable	
			1.3 Drug and toxin-induced	
			1.4 PAH associated with diseases	
			1.5 PAH long-term responders to CCB	
			1.6 PVOD	
			1.7 Persistent PH of newborn	
2 – Left heart disease	Left heart disease	Isolated post-capillary or combined pre- and post-capillary	2.1 PH due to HFpEF	Therapy-associated cardiomyopathy, radiation valvular heart disease, accelerated coronary artery disease, ICI cardiomyopathy and myocarditis
			2.2 PH due to HFrEF	
			2.3 Valvular heart disease	
			2.4 Congenital	
3 – Chronic Lung Disease	Chronic lung disease and hypoxemia	Pre-Capillary	3.1 Obstructive lung disease	Radiation pneumonitis, therapy-associated pneumonitis, Busulfan-induced pulmonary fibrosis
			3.2 Restrictive lung disease	
			3.3 Mixed obstructive/restrictive lung disease	
			3.4 Hypoxia without lung disease	
			3.5 Developmental disorders	
4 – CTEPH	CTEPH	Pre-Capillary	4.1 CTEPH	Cancer-associated hypercoaguable state, PTTM
			4.2 Other PA obstructions	
5 – Multifactorial	Unclear and Multifactorial	Pre-capillary, isolated post-capillary, combined pre- and post-capillary	5.1 Hematologic disorders	MPN, multiple myeloma
			5.2 Systemic and metabolic disorders	
			5.3 Others	
			5.4 Complex congenital heart disease	

CCB, calcium channel blocker; CTEPH, chronic thromboembolic pulmonary hypertension; HFpEF, heart failure with preserved ejection fraction; HFrEF, heart failure with reduced ejection fraction; ICI, immune checkpoint inhibitor; MPN, myeloproliferative neoplasms; PA, pulmonary artery; PAH, pulmonary arterial hypertension; PH, pulmonary hypertension; PVOD, pulmonary venous occlusive disease; PTTM, pulmonary tumor thrombotic microangiopathy; WHO, World Health Organization.

**Fig. 1.**
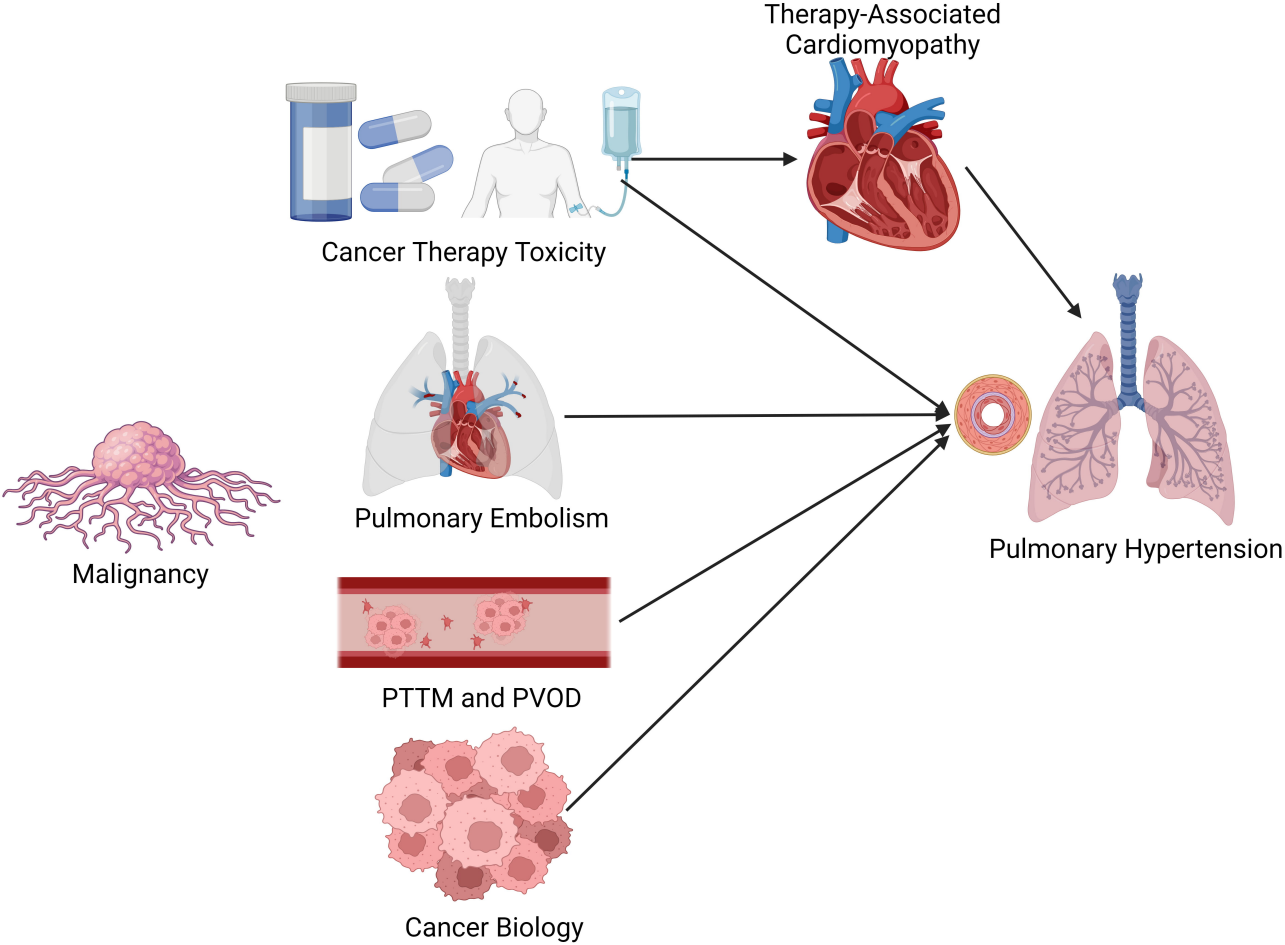
**Potential etiologies of pulmonary hypertension among patients with cancer**. Patients with cancer are at risk for developing pulmonary hypertension via several different etiologies including cancer therapy toxicity, hypercoaguability leading to increased risk of pulmonary embolism and tumor biology. PTTM, pulmonary tumor thrombotic microangiopathy; PVOD, pulmonary venous occlusive disease.

Pulmonary hypertension among patients with cancer is multifactorial and involves several pathophysiologic mechanisms that include the entire spectrum of hemodynamic and clinical characteristics of PH [11, 12]. Cancer therapies may cause group 1 PH via various mechanisms or cause group 2 or 3 PH due to cardiac and pulmonary toxicity, respectively (Fig. 1). Additionally, cancer is a prothrombotic state and is associated with an increased risk of venous thromboembolism (VTE) including pulmonary embolism and potentially the development of chronic thromboembolic pulmonary hypertension (CTEPH) in these patients [13]. Certain types of malignancies may be associated with the development of PH themselves, including myeloproliferative neoplasms (group 5) [14, 15]. We have reviewed mechanisms, diagnostic approach, and management for cancer therapy-related PH and RV dysfunction.

## 2. Pulmonary Hypertension in Cancer

Cancer and PH often coexist and may share similarities in underlying pathophysiologic processes [16]. In a multi-center registry study of patients with PH, 14.5% of patients with pre-capillary PH developed cancer during follow-up [17]. The prevalence of PH and its prognostic implications in cancer varies in the literature by cancer-type and the modality used to assess for PH. In one study of patients with lung cancer, 22.5% of patients had a pulmonary artery (PA) to aorta ratio of >1 (a strong predictor for PH) and was associated with decreased overall survival [18]. PH is commonly associated with myeloproliferative neoplasm (MPNs), a group of clonal hematopoietic stem cell disorders with an approximate prevalence of 33 [19, 20]. Among patients with newly diagnosed multiple myeloma (MM), the prevalence of PH was found to be 12.7% in one retrospective study [21].

The pathophysiology of PH in patients with cancer is multifactorial. Group 1 PH among patients with cancer can occur due to pulmonary venous occlusive disease (PVOD) and cancer therapy-associated PAH [22]. Though grouped together in group 1 PH, the pathophysiology of PVOD and PAH differs. PVOD is associated with pulmonary venous pathology while PAH affects the arterioles. Both PVOD and PAH present with group 1 PH but the diagnosis and management are different and out of the scope of this current review [23]. Patients with cancer may develop group 2 PH due to left sided heart disease from cancer therapy-associated cardiomyopathy, radiation-associated valvulopathy, and progression of underlying cardiovascular disease due to common pathophysiologic mechanisms that cancer and cardiovascular disease share in common [2, 6, 24, 25]. Cancer therapies, including busulfan, cyclophosphamide, bleomycin, immune checkpoint inhibitors and thoracic radiation therapy, have been associated with pulmonary toxicity and may increase the risk of group 3 PH in these patients [26, 27]. However, the incidence and prevalence of group 3 PH in patients with cancer therapy-associated pulmonary toxicity is not well characterized. Cancer is a prothrombotic state and patients with malignancy are at high risk of VTE, including pulmonary embolism (PE) and the subsequent development of CTEPH [28]. Indeed, cancer is a prevalent comorbidity among patients with CTEPH [13, 29, 30]. One study of patients with CTEPH reported a prevalence of cancer of 17% with breast and gastrointestinal cancers being the most common [13]. Group 5 PH has been described among patients with myeloproliferative neoplasms (MPN). Among patients with MPNs and pre-capillary PH, CTEPH and group 5 PH were the most common etiologies of PH [14]. Other rare etiologies of PH in patients with cancer include pulmonary tumor thrombotic microangiopathy (PTTM) and PVOD, which are almost always fatal [31, 32, 33].

Cancer therapy-related PH has been described to occur after the use of several cancer treatments and is an increasingly recognized cardiotoxicity [22]. Cancer therapeutics are thought to cause PH via different mechanisms including off-target inhibition of tyrosine kinases leading to vasoconstriction and smooth muscle proliferation, the development of PVOD, or left heart dysfunction in the setting of cardiomyopathy [22].

## 3. Cancer Therapies Associated with Pulmonary Hypertension and RV Dysfunction 

### 3.1 Alkylating Agents 

**Fig. 2.**
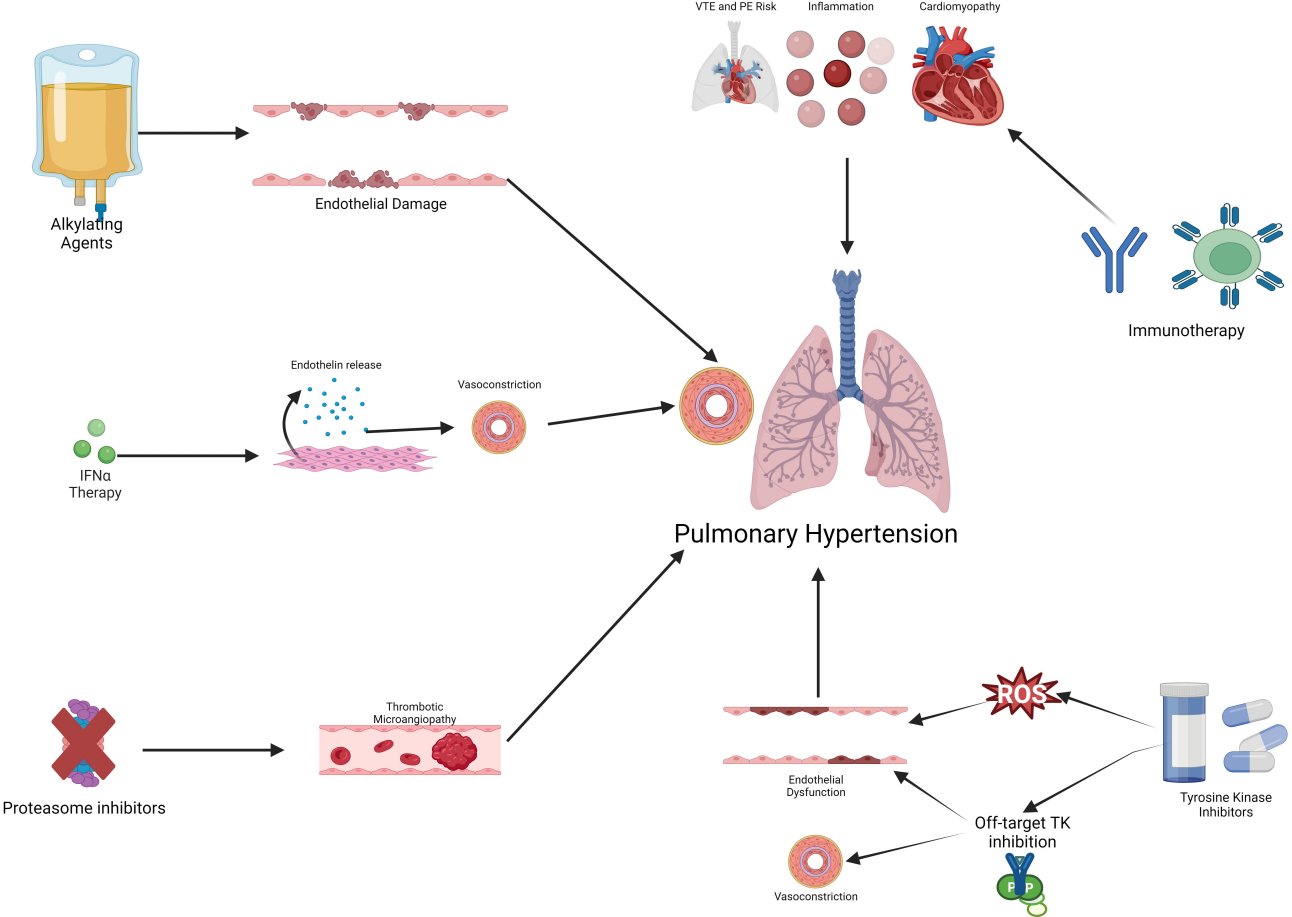
**Mechanisms of cancer therapy-associated pulmonary hypertension**. Cancer therapy can cause pulmonary hypertension via several possible mechanisms including endothelial damage and dysfunction, off-target TK inhibition, systemic inflammation, and inducing a pro-thrombotic state. IFNα, interferon-α; PE, pulmonary embolism; ROS, reactive oxygen species; TK, tyrosine kinase; VTE, venous thromboembolism.

Conventional chemotherapeutics have been implicated in the development of PAH (Fig. 2) [22]. One class of conventional chemotherapy implicated in PAH are alkylating agents, which include but are not limited to cyclophosphamide, melphalan, busulfan, and mitomycin-c [34]. These agents are often used to treat hematologic and solid malignancies. Several studies have implicated alkylating agents in the development of PAH, particularly PVOD which predominantly has pulmonary venous involvement [34]. In analysis of a French PH registry and systemic literature review, 37 cases of chemotherapy-induced PVOD were identified of whom 43.2% were treated with cyclophosphamide and 24.3% with mitomycin-c. Additionally, this study also showed rodents (mice, rats and rabbits) treated with cyclophosphamide had pathologic changes including medial hypertrophy of pulmonary arteries and pulmonary vein thickening [34]. Another study identified 7 patients with PVOD in a French PH registry after treatment with mitomycin-c for anal cancer [35]. Administration of mitomycin-c in rats led to elevated pulmonary artery pressures and major remodeling of small pulmonary veins [35]. The mechanism of PVOD after treatment with alkylating agents is not well characterized but may be due to vascular endothelial damage in pulmonary veins [35]. Interestingly, there may be a sex component given the disproportionate number of females with PVOD after mitomycin-c (6/7) despite a 2:1 male to female ratio for anal cancer among the French population [35]. Further studies are needed to better understand underlying mechanisms and delineate the risks and management of PH after alkylating chemotherapy treatment.

### 3.2 Interferon Therapy 

The interferons are a family of proteins that have important roles as extracellular messengers and are responsible for antiviral, antiproliferative, immunomodulatory activities [36]. The use of recombinant interferon-α is recommended as one option for cytoreduction in patients with MPNs, including polycythemia vera and essential thrombocytosis [37]. PH due to interferon therapy in malignancies has been described in the literature. In one study of 13 patients treated with interferon therapy (12 for chronic myeloid leukemia (CML) and 1 for melanoma), 2 patients (15%) developed PH [38]. In another study of patients with pre-capillary PH in a French PH registry, 53 patients with prior interferon exposure were identified. Of those patients, 48 had PH diagnosis after exposure to interferon and 16 patients were treated with interferon after the diagnosis of PH was made. Of the 16 patients with interferon treatment and known PH, 11 patients had an increase in PVR of more than 20% and in 5 patients who stopped interferon therapy had improvement in PH [39]. *In vitro* studies have suggested that interferon treatment may induce PH via increase release of endothelin-1 from pulmonary artery smooth muscle cells leading to vasoconstriction [40]. Additionally, interferon may induce PH by increasing pulmonary vascular permeability through thromboxane B cascade activation [41].

### 3.3 Proteasome Inhibitors

Proteasome inhibitors (including bortezomib and carfilzomib) are commonly used for the treatment of MM and are considered the backbone of combination therapy for MM [42]. Proteasome inhibitors have been associated with cardiovascular toxicity, including heart failure, arrhythmias, and acute myocardial infarction [42]. Several case reports have described PAH and RV failure with carfilzomib use [43, 44, 45]. However, conflicting pre-clinical data exists that suggest that proteasome inhibitors may ameliorate PH in animal models [46, 47]. One potential mechanism for the development of PH unique to proteasome inhibitors is thrombotic microangiopathy that has been described to occur in pulmonary microvasculature [48]. Further research is needed to describe the incidence of PH among patients treated with proteasome inhibitors and elucidate potential mechanisms of PH.

## 4. Tyrosine Kinase Inhibitors and Pulmonary Arterial Hypertension

Tyrosine kinases are a diverse group of enzymes that are important in normal cellular communication, homeostasis, proliferation, and signal transduction and have been implicated in oncogenesis in various tumors and malignancies [49, 50]. The development of tyrosine kinase inhibitors (TKIs) has revolutionized therapy paradigms for a broad range of hematologic and solid tumor malignancies, including but not limited to CML, chronic lymphocytic leukemia (CLL), non-small cell lung cancer (NSCLC), gastrointestinal stromal tumors (GIST), melanoma, and colorectal cancer [51]. Despite their relatively specific mechanism of action against tumor progression, TKIs may cause cardiotoxicity mainly through inhibition of tyrosine kinases not involved in oncogenesis [3]. The development of PH has been a well-documented toxicity of certain TKIs [52].

### 4.1 Dasatinib

Dasatinib is a TKI used in the treatment of CML or Philadelphia chromosome-positive acute lymphoblastic leukemia (ALL) [53]. The development of PH, particularly PAH, has been described among patients with CML or Philadelphia chromosome-positive ALL treated with dasatinib with an incidence of 0.45% to 12%, depending on the modality used for PH diagnosis and definition of PH [54, 55, 56]. In a randomized clinical trial of patients with CML treated with dasatinib compared with imatinib, patients on dasatinib had a 5% prevalence of PH compared with 0.4% of patients on imatinib [57]. A retrospective study of 451 CML patients on dasatinib who underwent transthoracic echocardiography (TTE) found that 56 (12%) of patients had an elevated right ventricular systolic pressure (RVSP) of >40 mmHg [55]. In another of study of 243 patients treated with dasatinib, 12.3% had RVSP >40 mmHg after a median follow-up of 27 months [58]. The results of this study showed an association between pericardial effusion, cardiopulmonary comorbidities, and dasatinib as 3rd line agent (vs 1st line) were associated with PH [58]. Dasatinib-induced PAH is typically a chronic process, with a median delay from drug initiation to diagnosis of 34 months [54]. Dasatinib-associated PH appears to be reversible in some patients after cessation of dasatinib [59, 60, 61]. In one study of the 56 patients with elevated RVSP on TTE after dasatinib initiation, 97% of patients had a decrease or normalization of RVSP after cessation of dasatinib [55]. Also in a registry of RHC-confirmed cases of dasatinib-associated PAH, drug cessation was associated with significant improvements in New York Heart Association (NYHA) class, walk distance, and PVR; however, PAH persisted in 37% of patients [62]. Management of dasatinib-induced PAH has not been thoroughly investigated though case reports have described improvement of PAH with traditional PAH therapy, including sildenafil and endothelin antagonists [63, 64].

There are a variety of proposed mechanisms, including dasatinib’s broad activity against off-target kinases including c-Src kinase, which is important in vascular smooth muscle proliferation [65, 66]. In rat models of PAH, chronic treatment with dasatinib led to exaggerated response to chronic hypoxia and was associated with pulmonary endothelial cell dysfunction [67]. The same study found that dasatinib, but not imatinib, induced apoptosis of human pulmonary endothelial cells *in vitro* via production of reactive oxygen species, independent of Src kinase inhibition [67].

### 4.2 Bosutinib and Ponatinib

Other TKIs have also been associated with PAH, though less is known about the mechanism and prevalence. Bosutinib and ponatinib are also TKIs used for treating CML and have been classified by European guidelines as having “possible” associations with PAH and may exert toxicity via off-target inhibition of Src protein kinase [8, 52, 68]. Case reports have described worsening PH after transitioning from dasatinib to bosutinib [69, 70, 71]. Similarly, case reports describe the occurrence of PH after the initiation of ponatinib therapy [72]. In a study utilizing human umbilical vein endothelial cells (HUVECs) *in vitro* suggested that ponatinib may induce an inflammatory phenotype and reduces endothelial nitric oxide synthase (*eNOS*) expression which may provide a pathophysiologic explanation of PH in ponatinib use [73].

### 4.3 Other Tyrosine Kinase Inhibitors

Tyrosine kinase inhibitors are a diverse group of medications with intended tyrosine kinase targets but also target unrelated tyrosine kinases which can cause adverse effects, including PH. Due to the heterogeneity of TKIs, it is challenging to discern if on-target or off-target effects are responsible for the development of PH. The epidermal growth factor receptor (EGFR) signaling has been implicated in the survival and proliferation of pulmonary artery smooth muscle cells (PASMCs) [74]. One study investigated the effect of EGFR inhibitors, including lapatinib, gefitinib, and erlotinib on rat and mouse models of PH [75]. While gefitinib and erlotinib led to improved hemodynamic and right ventricular function, lapatinib did not and lead to worsening PH. Similarly, in a small study of 27 patients treated with lapatinib showed an increase of pulmonary artery systolic pressure (PASP) on TTE after treatment [76]. Unlike erlotinib and gefitinib, lapatinib inhibits human epidermal growth factor receptor 2 (HER2) that may be responsible for the development of PH though further studies are needed [77]. Another TKI that has been associated with PH is ruxolitinib, a Janus kinase (JAK) 1/JAK2 inhibitor used in the treatment of myelofibrosis and polycythemia vera. One case report described a patient with myelofibrosis treated with ruxolitinib and panobinostat that developed pre-capillary PH on RHC that was reversed upon cessation of therapy [78]. However, myelofibrosis itself is associated with PH [14, 15, 19]. Additionally, other studies have suggested improvement in myelofibrosis-associated PH after treatment with ruxolitinib [79, 80]. In a rat model of CTEPH, treatment with ruxolitinib led to reduced pulmonary vascular remodeling and reduced right ventricular systolic pressure [81]. This is of clinical relevance since CTEPH is one of the most common etiologies of PH among patients with MPN [14]. Therefore, the role of JAK/signal transducer and activator of transcription (STAT) inhibition in the treatment of MPN-associated is fertile ground for further investigation.

## 5. Immunotherapy and Pulmonary Hypertension

Immunotherapy, including immune checkpoint inhibitors (ICI) and chimeric antigen receptor (CAR) T-cell therapy, have revolutionized the treatment paradigm for a wide range of cancers [82]. ICI work by targeting the programmed-cell-death-1 protein (PD-1), PD-1 ligand (PD-L1), and cytotoxic T-lymphocyte-associated antigen 4 (CTLA-4), which allows the patient’s own T-cells to target tumors [83]. CAT-T cell therapy are derived from the patient’s or donor T-cells and are engineered to target cancer antigens [84]. However, these therapies have been associated with immune-mediated cardiotoxicity including cardiomyopathy, myocarditis, accelerated atherosclerosis and increased VTE risk [85, 86, 87, 88]. PH is an under recognized cardiotoxicity that has been associated with cancer immunotherapy.

In a pharmacovigilance study, 42 PH (including 11 PAH, 1 PVOD) were identified of which half of cases were associated with nivolumab use [89]. These cases occurred a median of 77.0 days from initiation of therapy; 31% were fatal [89]. In a study of 59 patients with lung cancer treated with nivolumab, there was an increase of pulmonary artery to aorta diameter, a marker of PH, on computed tomography (CT) imaging from 0.82 to 0.87 (*p* < 0.001) [90]. Similarly, in a study of 117 of patients with hepatocellular carcinoma treated with atezolizumab (an ICI) and bevacizumab (anti-vascular endothelial growth factor (VEGF) monoclonal antibody), there was an increase in mean pulmonary artery diameter to aorta ratio on CT imaging after treatment (0.76 vs 0.79 mm, *p* < 0.001) [91].

It is important to note that these studies looked at surrogate markers of PH and did not include invasive hemodynamic data from RHC, which is the gold standard for the diagnosis of PH. Therefore, the hemodynamic characterization of ICI-associated PH is unclear and may be multifactorial given that ICI may cause pneumonitis (leading to group 3 PH), cardiomyopathy (leading to group 2 PH), as well as potential direct effect on pulmonary vasculature (group 1 PH). While cardiotoxicity, including cardiogenic shock, arrhythmias, and cardiomyopathy, have been described with CAR-T cell therapy, the incidence of PH is not well characterized or described in the literature [92, 93]. Further studies, especially those with RHC data, are needed to better characterize PH among patients treated with ICI. Cancer therapies associated with PH and potential mechanisms of action are summarized in Table 2 (Ref. [35, 40, 41, 48, 65, 66, 67, 73, 91]).

**Table 2.** **Cancer therapy-associated pulmonary hypertension and potential mechanisms**.

Cancer therapy	Mechanisms of pulmonary hypertension and RV dysfunction	Reference number
Alkylating agents (cyclophosphamide, melphalan, busulfan, mitomycin-c)	Endothelial damage, pulmonary venous remodeling	[35]
Interferon therapy	Endothelin-1 release mediated vasoconstriction, increased pulmonary vascular permeability via thromboxane B cascade activation	[40, 41]
Proteasome inhibitors	Thrombotic microangiopathy	[48]
Dasatinib	c-Src kinase inhibition leading to vascular smooth muscle cell proliferation, apoptosis of pulmonary endothelial cells via production of reactive oxygen species	[65, 66, 67]
Bosutinib and ponatinib	Reduced *eNOS* expression	[73]
Immune checkpoint inhibitors	Unclear mechanism, may be multifactorial (group 3 PH from pneumonitis, group 2 PH from left sided cardiomyopathy, increased inflammation)	[91]

*eNOS*, endothelial nitric oxide synthase; RV, right ventricular; PH, pulmonary hypertension.

## 6. Cancer-Therapy Associated Right Ventricular Dysfunction

Right ventricular dysfunction is associated with PH but can also occur in the absence of PH [94]. While left ventricular (LV) dysfunction and cardiomyopathy are well-known risks of cancer-therapy, these agents can also cause RV failure and cardiomyopathy [95, 96].

### 6.1 Anthracyclines

Anthracycline-based chemotherapies are commonly used to treat a wide range of cancers, including breast, hematologic and other solid tumors [6]. In a study of 30 patients with breast cancer treated with trastuzumab and anthracycline, 10% had concomitant RV dysfunction [97]. Another study of 155 patients with cancer therapy-associated cardiotoxicity (75% of whom received anthracycline-based therapy), RV free-wall longitudinal strain (RVFWLS), as assessed on echocardiography, allowed the identification of subclinical RV dysfunction [98]. One study involving cardiac magnetic resonance imaging (cMRI) of patients with breast cancer treated with anthracycline-based therapies showed decreased RV mass-index and cardiomyocyte mass after therapy and increased RV extracellular volume corresponding to increased interstitial fibrosis and RV atrophy [99]. The RV dysfunction, as evidenced by a reduced RV ejection fraction (RVEF), was reached nine months after the initiation of anthracycline-based cancer therapy. In contrast to LV dysfunction, RV dysfunction did not recover after completion of therapy suggesting that RV dysfunction may be less reversible than LV dysfunction after anthracycline chemotherapy [99]. RV dysfunction was noted in 21.7% of patients in a study of 249 patients with cancer who underwent cMRI for suspected anthracycline-related cardiomyopathy and was associated with increased risk of major adverse cardiovascular events (MACE) though this was not significant after multivariable adjustment [100].

### 6.2 Trastuzumab 

Trastuzumab is a monoclonal antibody that targets erbB-2 and erbB-3 receptors and is used to treat HER2-positive breast cancer. Trastuzumab has been associated with cardiomyopathy and LV dysfunction with its deleterious effects on cardiac function being compounded if used in combination with anthracyclines [6]. In a study of 41 patients treated with trastuzumab who underwent cMRI showed that treatment with trastuzumab was associated with a reduction of RVEF (58% pre-treatment vs 55% 6 months post-treatment, *p* < 0.001) though RVEF tended to recover 18 months after treatment [101]. Among 101 patients treated with trastuzumab, RVFWLS predicted cardiotoxicity [102].

### 6.3 Immune-Checkpoint Inhibitors

Right ventricular dysfunction has been described after treatment with ICI [103, 104]. In a study of 24 patients treated with ICI who had baseline and follow-up TTEs, there was a significant reduction in RV function as measured by RV free wall longitudinal strain after a median of 85 days of ICI treatment. In this study, most patients were treated with ICI for lung cancer (92%), 25% were treated with nivolumab and 29% with pembrolizumab [103]. Additionally, RV myocarditis has been described in one case report after ICI therapy [104].

## 7. Interventional Cardiology Tools for Management of Pulmonary Hypertension in Patients with Cancer

Advances in catheter-based techniques have flourished in the past couple of decades and have been applied to the treatment and management of PH [105]. Monitoring of pulmonary artery pressure via implantable sensors (i.e., CardioMEMS, Abbott) have been developed for the management of patients with heart failure [106]. These devices have been studied in small studies of patients with PH, including PAH, and suggest that pulmonary artery pressure monitoring may be useful in these patients though larger studies are lacking [107, 108]. Among patients with cancer, data on outcomes and usefulness of pulmonary artery pressure monitoring in PH or heart failure are lacking. In one case report, pulmonary artery pressure monitoring using CardioMEMS system was used to guide therapy in a patient with anthracycline cardiomyopathy undergoing CAR T-cell therapy [109]. This suggests that pulmonary artery pressure monitoring may be a novel tool to monitor the development of therapy-associated PH or cardiotoxicity among high-risk patients, though studies are needed.

Cancer is a risk factor for VTE and subsequent development of CTEPH [13, 29]. Surgical pulmonary thromboendarterterectomy is standard of care for CTEPH [110]. In patients at prohibitive risk of surgery, as cancer patients often are, balloon pulmonary angioplasty (BPA) provides a therapeutic option [111]. Successfully treatment of CTEPH with BPA has been described in case reports [112, 113, 114]. However, larger studies are needed to assess efficacy and safety in patients with cancer.

## 8. Management of Cancer Therapy-Associated Pulmonary Hypertension and Future Directions

Patients with cancer receiving therapy associated with PH should be monitored carefully for the development of PH. While the current European Society of Cardiology (ESC) and European Respiratory Society (ERS) guidelines do not mention screening patients starting cancer therapy for PH, the current ESC cardio-oncology guidelines recommend echocardiographic evaluation if new symptoms of PH develop (shortness of breath, fatigue, etc.) [6, 8]. If the peak tricuspid regurgitation velocity (TRV) of ≤2.8 m/s and no other echocardiographic signs of PH (dilated inferior vena cava, right ventricular dilation or hypertrophy, RV/LV ratio >1) then the probability of PH is considered to be low [8]. A peak TRV of >3.4 m/s or echocardiographic signs of PH in patients with cancer undergoing therapy should prompt further investigation and surveillance, especially if these findings are new compared to before the initiation of cancer treatment [6]. The definitive diagnosis of PH requires RHC and should be considered among patients with findings on echocardiography suggestive of PH. Interruption or dose reduction of dasatinib is sometimes recommended among patients with newly diagnosed PH while on therapy with monitoring of peak TRV every 3 months after dose reduction [6, 54].

**Fig. 3.**
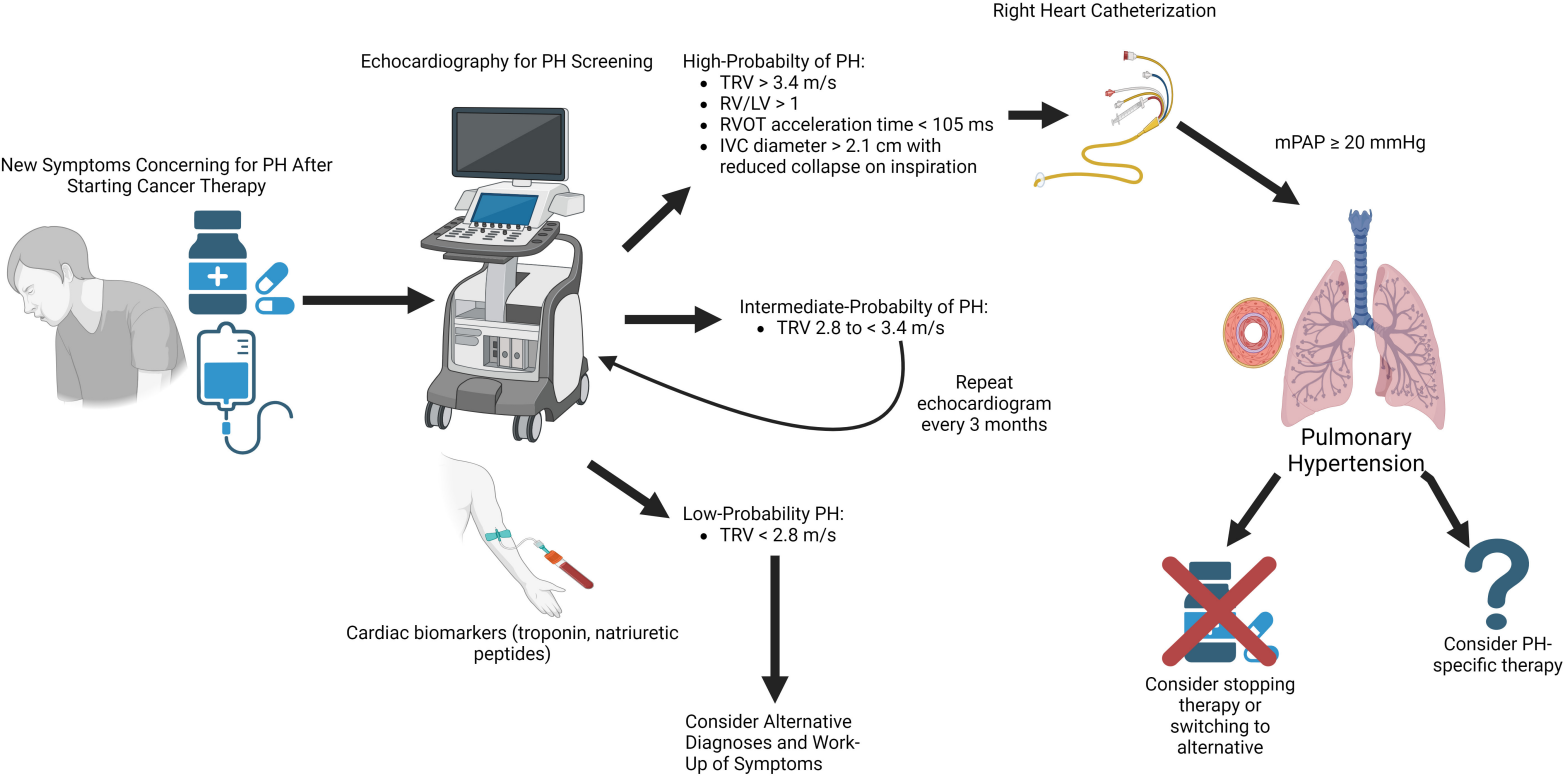
**Proposed algorithm for diagnosis and surveillance of cancer therapy-associated pulmonary hypertension**. Symptosm concerning for PH after initiation of cancer therapy should be investigated with blood work and echocardiography. Patients with findings on echocardiography with high-probability of PH should be considered for right heart catheterization for diagnosis of PH. Strong evidence for the safety and efficacy of PH-specific therapy is lacking but can be considered for patients with right heart catheterization-proven PAH. IVC, inferior vena cava; LV, left ventricle; mPAP, mean pulmonary artery pressure; PAH, pulmonary arterial hypertension; PH, pulmonary hypertension; RV, right ventricle; RVOT, right ventricular outflow tract; TRV, tricuspid regurgitation maximum velocity.

It is important to note that the ESC cardio-oncology guidelines primarily address the role of surveillance for dasatinib-associated PH as compared to other therapies. However, given the paucity of data of PH in cancer therapy, extrapolation to other cancer therapies is necessary but further investigation is needed. Additionally, the recommendations of the guidelines are based on expert consensus due to the lack of data currently available [6]. Whether PH-specific therapies, including phosphodiesterase inhibitors, endothelin receptor antagonists, and prostacyclins, are efficacious and improve outcomes in cancer-associated PH is yet to be examined in rigorous clinical investigations but merits further study. Epidemiologic studies to characterize and identify risk factors for PH among patients with cancer undergoing treatment are needed. Prospective studies on PH-specific therapies on cancer therapy-associated PH outcomes are also crucially needed (Fig. 3).

## 9. Conclusions

Cancer therapy-associated PH and RV dysfunction is an underappreciated form of cardiovascular toxicity from conventional and novel cancer therapeutics. Patients with cancer are also at risk of PH from multifactorial etiologies including cancer therapy, thrombosis and cancer-specific pathology. Future studies are needed to better characterize PH in patients with cancer, including investigations involving RHC for better characterization of hemodynamic classification of PH in cancer. Additionally, among patients treated with ICI, response of PH to immunosuppression should be investigated. Patients with cancer being treated with high-risk therapy should be monitored closely for the development of PH. Additionally, novel interventions, including transcatheter devices and pressure sensor monitoring, for PH should be studied in patients with cancer in order to determine their utility in monitoring, preventing and managing cancer therapy-associated PH. Understanding mechanisms of PAH induced by both historical and modern cancer treatment regimens may improve our understanding of other phenotypes of PAH, in addition to yielding insights into potential novel treatment strategies that can be used to treat both traditional forms of PAH and within the cardio-oncology population.
